# Factors associated with urinary and double incontinence in a geriatric post-hip fracture assessment in older women

**DOI:** 10.1007/s40520-021-02046-z

**Published:** 2022-01-04

**Authors:** Aino Tuulikki Hellman-Bronstein, Tiina Hannele Luukkaala, Seija Sinikka Ala-Nissilä, Minna Anneli Kujala, Maria Susanna Nuotio

**Affiliations:** 1 Division of Geriatric Medicine, Department of Clinical Medicine, University of Turku, Turku, Finland; 2 Research, Development and Innovation Center, Tampere University Hospital, Tampere, Finland; 3 Health Sciences, Faculty of Social Sciences, Tampere University, Tampere, Finland; 4 Department of Obstetrics and Gynecology, Turku University Hospital, Turku, Finland; 5 Department of Clinical Medicine, University of Turku, Turku, Finland; 6 Department of Geriatric Medicine, Seinäjoki Central Hospital, Seinäjoki, Finland; 7 Research Services and Department of Clinical Medicine, Turku University Hospital, Turku, Finland; 8 Welfare Division, City of Turku, Turku, Finland

**Keywords:** Urinary incontinence, Double incontinence, Hip fracture, Frailty

## Abstract

**Background:**

Incontinence and hip fractures are common in older people, especially women, and associated with multiple adverse effects. Incontinence is a risk factor for falls.

**Aims:**

We aimed to investigate the prevalence of urinary (UI) and double incontinence (DI, concurrent UI and faecal incontinence), and to identify factors associated with UI and DI 6 months post-fracture.

**Methods:**

A prospective real-life cohort study was conducted consisting of 910 women aged ≥ 65 who were treated for their first hip fracture in Seinäjoki Central Hospital, Finland, between May 2008 and April 2018. Continence status was elicited at baseline and 6 months postoperatively at our geriatric outpatient clinic where all participants underwent a multidisciplinary comprehensive geriatric assessment (CGA) consisting of an evaluation of cognition, nutrition, mood, mobility, and functional ability.

**Results:**

At baseline, 47% of the patients were continent, 45% had UI and 8% had DI, and at follow up, 38%, 52%, and 11%, respectively. The mean age of the patients was 82.7 ± 6.8. Both UI and DI were associated with functional disability and other factors related to frailty. The associations were particularly prominent for patients with DI who also had the worst performance in the domains of CGA. We identified several modifiable risk factors: depressive mood (odds ratio [OR] 1.81; 95% confidence interval [CI] 1.16–2.84) and constipation (OR 1.48, 95% CI 1.02–2.13) associated with UI and, late removal of urinary catheter (OR 2.33, 95% CI 1.31–4.14), impaired mobility (OR 2.08, 95% CI 1.05–4.15), and poor nutrition (OR 2.31, 95% CI 1.11–4.79) associated with DI.

**Conclusions:**

This study demonstrates a high prevalence of UI and DI in older women with hip fracture and modifiable risk factors, which should be targeted in orthogeriatric management and secondary falls prevention. Patients with DI were found to be an especially vulnerable group.

**Supplementary Information:**

The online version contains supplementary material available at 10.1007/s40520-021-02046-z.

## Introduction

Urinary incontinence (UI), defined as any reported involuntary loss of urine [1], has long been recognized as a geriatric syndrome associated with adverse patient outcomes, such as functional decline, decreased quality of life, depression, institutionalization, and mortality. UI is more common in women than in men, and prevalence increases with advancing age. Nearly 40% of women older than 60 years and nearly 80% of women residing in long-term care have UI. Despite high prevalence, UI remains under-reported and poorly managed [2–4].

Double incontinence (DI) is defined as the concurrent UI and faecal incontinence (FI) [5]. DI, the most severe form of pelvic floor dysfunction, is associated with greater decline in quality of life than UI or FI in isolation. As with UI, DI is more common among women and associated with advancing age. Risk factors for DI include high comorbidity, frailty, depression, and limited mobility. The prevalence of DI in community-dwelling adults has ranged between 2.5 and 14.5% in prior studies. In a large cross-sectional study of over 64,000 community-dwelling older women, the prevalence of DI was 7% [6–8]. The prevalence of DI is higher and estimated around 33–65% in patients residing in long-term care and, it becomes more common with increasing time after admission. DI is associated with UI, functional and cognitive deficits, comorbidities, and advancing age in these patients [9, 10].

UI is known to be associated with falls in older adults, especially women, and both are associated with frailty. Frailty is a multidimensional syndrome defined as diminished physiologic reserve and function with increased vulnerability to minor stressors and risk for adverse health outcomes [11–14]. UI is twice as common among frail older adults as compared to the robust, and underlying causes of incontinence in this group are diverse and often related to factors outside the urinary tract; namely, functional and cognitive decline, comorbidity, and polypharmacy [15].

Research on the connection between DI and falls or frailty is scarce. In one study of older community-dwelling Brazilian women, falls experienced during the previous year were found to be a risk factor for incident DI [16]. Moreover, FI has been associated with falls [17] and mortality after a hip-fracture in older adults [18]. Therefore, our hypothesis is that DI is associated with poor outcomes after a hip fracture.

The aim of this study was to investigate the prevalence and factors associated with UI and DI among older women with hip fracture. Moreover, we sought to examine associated factors separately for UI and DI, since we assumed that the DI group represented the frailest of the study population.

## Methods

### Study population

The study population comprised 1675 consecutive women aged ≥ 65 years who were treated for their first hip fracture in Seinäjoki Central Hospital, Finland, between May 2008 and April 2018. Seinäjoki Central Hospital is the only hospital providing acute surgical care in the Hospital district of Southern Ostrobothnia, which has a population of approximately 200,000. The patients were discharged from the acute hospital into primary care hospitals for rehabilitation after a median length of stay of 5 days (interquartile range [IQR] of 2–8 days). The only exclusion criteria in this study were pathologic and periprosthetic fractures. All the patients regardless of cognitive state, morbidity or functional ability or living arrangements were invited to participate.

### Data collection

The baseline data was collected during hospitalization by a trained geriatric nurse interviewing the patients or their representatives, and from patient medical records. In addition, follow-up data was collected at the outpatient clinic during the CGA visit in a median time of 6 months (IQR 4–6 months). The CGA was performed by a multidisciplinary team lead by a geriatrician or a trainee in geriatric medicine under her supervision. A geriatric nurse carried out the assessment of the domains with the selected tools. Both the patient and his or her next of kin or caregiver were invited. A physiotherapist’s examination preceded the geriatric assessment. Informed consent was obtained from all the patients or their representatives (legal guardian or next of kin). The study was approved by the Ethics Committee of the Hospital District of Southern Ostrobothnia.

### Variables and definitions

During the initial perioperative interview, continence status before the fracture was elicited from the patients or their representatives. UI was defined as any reported involuntary loss of urine, and FI as any reported involuntary loss of faeces, respectively. Patients with both symptoms were deemed to have DI. Data on demographics, number of prescribed medications, diagnosis of cognitive disorder (yes or no), mobility, and living arrangements were collected. Independent mobility was defined as being able to ambulate independently without personal assistance. Living in an institution was defined as residing in a primary care hospital or a long-term care facility (LTCF) providing 24-h care.

The preoperative American Society of Anaesthesiologists (ASA) risk scores were used to assess general health at baseline. There are five classes: (1) healthy person, (2) mild systemic disease, (3) severe systemic disease, (4) severe systemic disease that is a constant threat of life, and (5) a moribund person who is not expected to survive without an operation [19]. Nutritional status was assessed using Mini Nutritional Assessment Short Form (MNA-SF) [20] both during hospitalization and at follow up. For the purposes of our study, poor nutrition was defined as being at risk for malnutrition or being malnourished according to the MNA-SF. Fracture type and the removal or non-removal of urine catheter (UC) before discharge from the acute hospital care were extracted from the medical records. Categorization of the baseline variables is shown in Table 1.

**Table 1:** Distribution of the baseline indicators according to continence status (*N* = 910)

	*N*	Continent		Urinary incontinent		Double incontinent		
		*n* = 343		*n* = 469		*n* = 98		
		*n*	(%)	*n*	(%)	*n*	(%)	*p*
Age								< 0.001
65–79	273	133	(39)	113	(24)	27	(28)	
80–89	504	177	(52)	274	(58)	53	(54)	
≥ 90	133	33	(10)	82	(18)	18	(18)	
Fracture type								0.038
Intracapsular	564	230	(67)	276	(59)	58	(59)	
Extracapsular	344	112	(33)	193	(41)	39	(40)	
Not known	2	1	(0)	0	(0)	1	(1)	
ASA								0.013
1–2	154	75	(22)	71	(15)	8	(8)	
3–5	742	264	(77)	390	(83)	88	(90)	
Not known	14	4	(1)	8	(2)	2	(2)	
Number of regularly taken medications								0.001
< 4	171	84	(25)	80	(17)	7	(7)	
4–10	584	211	(62)	301	(64)	72	(74)	
> 10	155	48	(14)	88	(19)	19	(19)	
Diagnosis of cognitive disorder								< 0.001
No	682	307	(90)	320	(68)	55	(56)	
Yes	227	36	(11)	148	(32)	43	(44)	
Not known	1	0	(0)	1	(0)	0	(0)	
Mobility								< 0.001
Independent	567	270	(79)	267	(57)	30	(31)	
Non-independent	340	71	(21)	201	(43)	68	(69)	
Not known	3	2	(1)	1	(0)	0	(0)	
Living arrangements								< 0.001
Home	707	309	(90)	353	(75)	45	(46)	
Institution	199	34	(10)	112	(24)	53	(54)	
Not known	4	0	(0)	4	(1)	0	(0)	
MNA-SF before hip fracture								< 0.001
Normal (12–14)	428	179	(52)	220	(47)	29	(30)	
Poor nutrition (< 12)	302	77	(22)	180	(38)	45	(46)	
Not known	180	87	(25)	69	(15)	24	(25)	
Removal of urine catheter								< 0.001
During hospital stay	530	228	(67)	263	(56)	39	(40)	
Later	372	113	(33)	200	(43)	59	(60)	
Not known	8	2	(1)	6	(1)	0	(0)	
Continence before fracture								< 0.001
Continent	431	268	(78)	149	(32)	14	(14)	
Urinary incontinent	406	71	(21)	282	(60)	53	(54)	
Double incontinent	73	4	(1)	38	(8)	31	(32)	

Differences (*p* value) between the continence groups were tested using Pearson Chi-square test or Fisher’s exact test

*ASA* American Society of Anesthesiologists -risk score, *MNA*-*SF* Mini Nutritional Assessment Short Form

At the outpatient clinic, different domains of the CGA were assessed using standardized and well-known measures [21]. Cognitive function was measured with the Mini Mental State Examination (MMSE) [22], depressive mood with the 15-item Geriatric Depression Scale (GDS-15) [23], and Instrumental Activities of Daily Living (IADL) by Lawton and Brody [24]. Continence status, constipation, and new falls after the fracture were elicited during the interview. The physiotherapist’s assessment of the patient’s physical performance included the Timed up and go-test (TUG) assessing mobility, balance, and risk of falls [25], the Elderly Mobility Scale (EMS) which represents patient’s mobility and ability to perform basic ADL (activities of daily living) [26], and grip strength as a measure of muscle strength. Grip strength was measured in the stronger hand with a Jamar dynamometer and defined as weakened if less than 16 kg [27]. The categorization of the variables used at the outpatient clinic is shown in Table 2.

**Table 2:** Distribution of the outpatient domains according to continence status (*N* = 910) after 6 months follow-up

	*N*	Continent		Urinary incontinent			Double incontinent			
		*n* = 343		*n* = 469			*n* = 98			
		*n*	(%)		*n*	(%)		*n*	(%)	*p*
MMSE										< 0.001
Normal (24–30)	323	164	(48)		144	(31)		15	(15)	
Abnormal (< 24)	555	176	(51)		308	(66)		71	(72)	
Not known	32	3	(1)		17	(4)		12	(12)	
IADL										< 0.001
No difficulties (8)	154	98	(29)		54	(12)		2	(2)	
Difficulties (0–7)	742	239	(70)		407	(87)		96	(98)	
Not known	14	6	(2)		8	(2)		0	(0)	
GDS-15										< 0.001
Normal (0–6)	703	301	(88)		345	(74)		57	(58)	
Depressed (> 6)	152	35	(10)		98	(21)		19	(19)	
Not known	55	7	(2)		26	(6)		22	(22)	
EMS										< 0.001
Normal (14–20)	596	285	(83)		284	(61)		27	(28)	
Abnormal (< 14)	263	49	(14)		159	(34)		55	(56)	
Not known	51	9	(3)		26	(6)		16	(16)	
TUG										< 0.001
Normal (1–2)	280	148	(43)		119	(25)		13	(13)	
Abnormal (3–5)	477	167	(49)		270	(58)		40	(8)	
Not known	153	28	(8)		80	(17)		45	(46)	
Grip strength, stronger hand										< 0.001
Normal (≥ 16 kg)	186	86	(25)		90	(19)		10	(10)	
Abnormal (< 16 kg)	448	144	(42)		258	(55)		46	(47)	
Not known	276	113	(33)		121	(26)		42	(43)	
MNA-SF										< 0.001
Normal (12–14)	371	179	(52)		180	(38)		12	(12)	
Poor nutrition (< 12)	524	158	(46)		285	(61)		81	(83)	
Not known	15	6	(2)		4	(1)		5	(5)	
Constipation										< 0.001
No	307	137	(40)		143	(31)		27	(28)	
Yes	354	94	(27)		220	(47)		40	(41)	
Not known	249	112	(33)		106	(23)		31	(32)	
New falls after fracture										0.017
No	665	271	(79)		327	(70)		67	(68)	
Yes	244	71	(21)		142	(30)		31	(32)	
Not known	1	1	(0)		0	(0)		0	(0)	

Differences between continence groups (*p* value) were tested using Pearson Chi-square test or Fisher’s exact test

*MMSE* mini mental state examination, *IADL* instrumental activities of daily living, *GDS*-*15* geriatric depression scale, *EMS* elderly mobility scale, *TUG* timed up and go –test, *MNA*-*SF* Mini Nutritional Assessment Short Form

### Statistical analysis

Distributions of the baseline characteristics between the patients who were continent and those who had UI or DI are shown in Table 1. Distributions of the outpatient domains between the respective groups are presented Table 2. Groupwise comparisons were performed using the Pearson’s Chi^2^ test or Fisher’s exact test.

Age- and multivariable-adjusted multinomial logistic regression analyses with odds ratios (OR) and 95% confidence intervals (CI) were conducted to examine the associations of both the baseline variables and outpatient domains with UI and DI at follow up using the continent group as a reference. IBM SPSS Statistics version 25.0 for Windows software (SPSS Inc. Chicago, Illinois) was used for statistical analyses. All tests were two-sided, and *p* values < 0.05 were considered statistically significant.

## Results

**Fig. 1:**
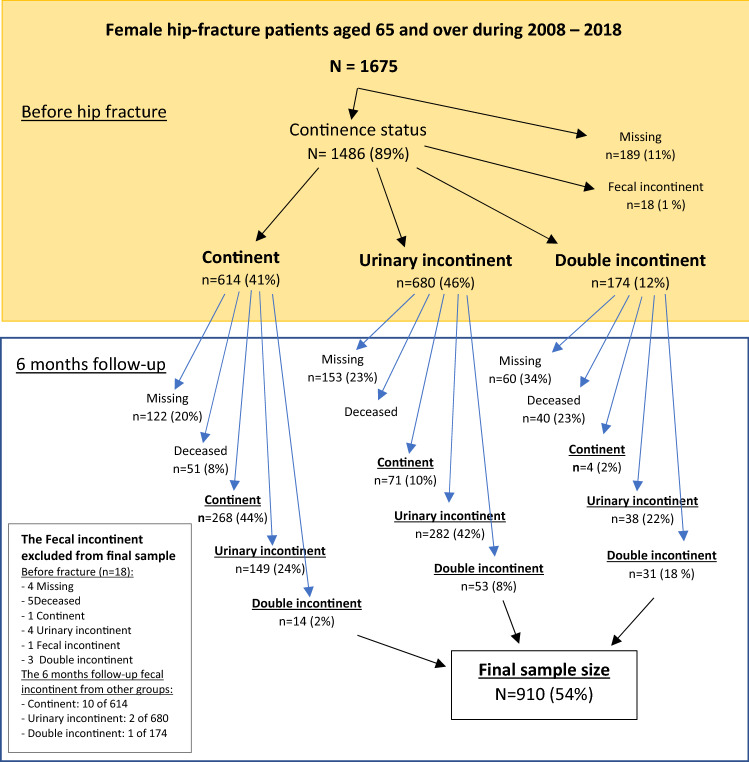
A flow chart of continence status in the study population

A total of 1675 participants were included in this study. The flow chart related to participant selection is shown in Fig. 1, whereas the participant characteristics are presented in Tables 1 and 2. The few patients (*n* = 31) with FI only were excluded from the final sample. The mean age of the patients was 82.7 ± 6.8. At baseline, 25% of the patients had a cognitive disorder, 22% lived in an institution, 33% had poor nutrition, and 37% could not ambulate independently.

Out of the 910 patients, 431 (47%) were continent, 406 (45%) had UI and 73 (8%) had DI at baseline, and at follow up, 343 (38%), 469 (52%) and 98 (11%), respectively. During the follow-up period, the continence status improved in 113 (12%) and deteriorated in 216 (24%) of the patients (Fig. 1).

In the age-adjusted univariate analyses, UI at follow up was associated with the following baseline variables: age over 80, ≥ 10 regular medications per day, diagnosis of cognitive disorder, non-independent mobility, living in an institution, poor nutrition, late removal of UC and previous UI or DI. In age-adjusted univariate analyses, DI at follow up was associated with the baseline variables including age between 80 and 89, ASA score higher than 2, ≥ 4 regular medications per day, diagnosis of cognitive disorder, non-independent mobility, poor nutrition, late removal of UC, and previous UI or DI (Table A1). Except for grip strength, all the domains of the CGA assessed at the outpatient clinic, observed constipation and new falls after fracture were significantly associated with both UI and DI at follow up in the age-adjusted univariate analyses (Table A2).

In the multivariable models of the baseline variables, UI at the follow-up visit was independently associated with age over 90, previous diagnosis of cognitive disorder and pre-fracture UI or DI while an independent association was observed between UI and difficulties in IADL, depressive mood, abnormal EMS, and self-reported constipation in the outpatient CGA. The respective results of the multivariable-adjusted analyses for associations with DI at follow up were non-independent mobility, living in an institution, late removal of UC and previous UI or DI at baseline, as well as difficulties in IADL, abnormal EMS, and poor nutrition observed in the outpatient CGA (Tables 3 and 4).

**Table 3:** Multivariable-adjusted associations of the baseline indicators with urinary or double incontinence after 6 months follow-up among hip fracture patients (*N* = 910) were analyzed using multinomial regression

	Urinary incontinent (*n* = 469)					Double incontinent (*n* = 98)				
	Multivariable-adjusted					Multivariable-adjusted				
	*n*	(%)	OR	(95% CI)	*p*	*n*	(%)	OR	(95% CI)	*p*
Age										
65–79	113	(24)	1.00			27	(28)	1.00		
80–89	274	(58)	1.22	(0.84–1.78)	0.291	53	(54)	0.76	(0.41–1.43)	0.402
≥ 90	82	(17)	**1.78**	**(1.01–3.13)**	**0.045**	18	(18)	1.16	(0.49–2.74)	0.737
Fracture type										
Intracapsular	276	(59)	1.00			58	(59)	1.00		
Extracapsular	193	(41)	1.20	(0.85–1.68)	0.298	39	(40)	1.23	(0.71–2.13)	0.455
ASA										
1–2	71	(15)	1.00			8	(8)	1.00		
3–5	390	(83)	1.00	(0.63–1.53)	0.918	88	(90)	1.21	(0.50–2.92)	0.679
Number of regularly taken medications										
< 4	80	(17)	1.00			7	(7)	1.00		
4–10	301	(64)	1.20	(0.79–1.83)	0.401	72	(74)	2.08	(0.84–5.15)	0.115
> 10	88	(19)	1.13	(0.64–2.00)	0.666	19	(19)	1.40	(0.48–4.09)	0.541
Diagnosis of cognitive disorder										
No	320	(68)	1.00			55	(56)	1.00		
Yes	148	(32)	**2.06**	**(1.28–3.23)**	**0.003**	43	(44)	1.31	(0.67–2.62)	0.443
Mobility										
Independent	267	(57)	1.00			30	(31)	1.00		
Non-independent	201	(43)	1.38	(0.90–2.13)	0.142	68	(69)	**2.08**	**(1.05–4.15)**	**0.037**
Living arrangements										
Home	353	(75)	1.00			45	(46)	1.00		
Institution	112	(24)	1.10	(0.65–1.87)	0.721	53	(54)	**2.55**	**(1.27–5.15)**	**0.009**
MNA-SF before hip fracture										
Normal (12–14)	220	(47)	1.00			29	(30)	1.00		
Poor nutrition (< 12)	180	(38)	1.09	(0.73–1.63)	0.663	45	(46)	1.52	(0.78–2.95)	0.216
Not known	69	(15)	0.71	(0.45–1.12)	0.141	24	(25)	1.44	(0.67–3.06)	0.348
Removal of urine catheter										
During hospital stay	263	(56)	1.00			39	(40)	1.00		
Later	200	(43)	1.36	(0.96–1.94)	0.088	59	(60)	**2.33**	**(1.31–4.14)**	**0.004**
Continence before fracture										
Continent	149	(32)	1.00			14	(14)	1.00		
Urinary incontinent	282	(60)	**5.76**	**(4.08–8.13)**	** < 0.001**	53	(54)	**11.80**	**(5.92–23.5)**	** < 0.001**
Double incontinent	38	(8)	**9.29**	**(3.12–27.6)**	** < 0.001**	31	(32)	**58.21**	**(16.8–201)**	** < 0.001**

Reference group was no incontinence (*n* = 343). Results are shown by odds ratios (OR) with 95% Confidence intervals (CI). Statistically significant (*p* < 0.05) ORs are in bold

*ASA* American Society of Anesthesiologists-risk score, *MNA*-*SF* Mini Nutritional Assessment Short Form

**Table 4:** Multivariable-adjusted associations of outpatient domains with urinary or double incontinence after 6 months follow-up among hip fracture patients (*N* = 910) were analyzed using multinomial regression

	Urinary incontinent (*n* = 469)					Double incontinent (*n* = 98)				
	Multivariable-adjusted					Multivariable-adjusted				
	*n*	(%)	OR	(95% CI)	*p*	*n*	(%)	OR	(95% CI)	*p*
Age										
65–79	113	(24)	1.00			27	(28)	1.00		
80–89	274	(58)	1.24	(0.85–0.81)	0.269	53	(54)	0.72	(0.37–1.40)	0.332
≥ 90	82	(17)	1.56	(0.89–2.74)	0.119	18	(18)	0.95	(0.39–2.31)	0.904
MMSE										
Normal (24–30)	144	(31)	1.00			15	(15)	1.00		
Abnormal (< 24)	308	(66)	1.12	(0.79–1.59)	0.537	71	(72)	1.25	(0.63–2.50)	0.528
IADL										
No difficulties (8)	54	(12)	1.00			2	(2)	1.00		
Difficulties (0–7)	407	(87)	**1.58**	**(1.00–2.51)**	**0.049**	96	(98)	**5.99**	**(1.28–28.0)**	**0.023**
GDS-15										
Normal (0–6)	345	(74)	1.00			57	(58)	1.00		
Depressed (> 6)	98	(21)	**1.81**	**(1.16–2.84)**	**0.009**	19	(19)	1.64	(0.83–3.25)	0.156
EMS										
Normal (14–20)	284	(61)	1.00			27	(28)	1.00		
Abnormal (< 14)	159	(34)	**2.00**	**(1.29–3.12)**	**0.002**	55	(56)	**4.55**	**(2.19–9.48)**	** < 0.001**
TUG										
Normal (1–2)	119	(25)	1.00			13	(13)	1.00		
Abnormal (3–5)	270	(58)	1.28	(0.88–1.86)	0.201	40	(8)	0.67	(0.30–1.50)	0.33
Not known	80	(17)	1.66	(0.82–3.35)	0.159	45	(46)	1.92	(0.65–5.70)	0.238
Grip strength, stronger hand										
Normal (≥ 16 kg)	90	(19)	1.00			10	(10)	1.00		
Abnormal (< 16 kg)	258	(55)	0.94	(0.63–1.41)	0.760	46	(47)	1.13	(0.52–2.48)	0.752
Not known	121	(26)	0.62	(0.37–1.04)	0.068	42	(43)	0.81	(0.33–1.96)	0.634
MNA-SF										
Normal (12–14)	180	(38)	1.00			12	(12)	1.00		
Poor nutrition (< 12)	285	(61)	0.94	(0.67–1.31)	0.705	81	(83)	**2.31**	**(1.11–4.79)**	**0.025**
Constipation										
No	143	(31)	1.00			27	(28)	1.00		
Yes	220	(47)	**1.48**	**(1.02–2.13)**	**0.038**	40	(41)	0.84	(0.44–1.60)	0.598
Not known	106	(23)	0.93	(0.57–1.52)	0.777	31	(32)	0.84	(0.38–1.86)	0.662
New falls after fracture										
No	327	(70)	1.00			67	(68)	1.00		
Yes	142	(30)	1.34	(0.94–1.90)	0.108	31	(32)	1.66	(0.94–2.93)	0.078

Reference group was no incontinence (*n* = 343). Results are shown by odds ratios (OR) with 95% Confidence intervals (CI). Statistically significant (*p* < 0.05) ORs are in bold

*MMSE* mini mental state examination, *IADL* instrumental activities of daily living, *GDS*-*15* geriatric depression scale, *EMS* elderly mobility scale, *TUG* timed up and go-test, *MNA*-*SF* Mini Nutritional Assessment Short Form

## Discussion

UI and DI were common in this large cohort study of older women with hip fracture. Both UI and DI were associated with functional disability and other factors related to frailty. The associations were particularly prominent for patients with DI. Moreover, patients with DI performed more poorly in the domains of CGA at follow up as compared to the patients with UI or without either condition suggesting that patients with DI represent a frailer patient population than those with UI alone. We identified several potentially modifiable risk factors, namely depressive mood and constipation associated with UI and, late removal of UC, impaired mobility, and poor nutrition associated with DI.

Half of the surviving patients had UI while every tenth patient had DI at the end of follow up. Our results were in line with previous studies on hip fracture patients [28, 29]. However, DI was slightly more common in our study than in the previous studies on community-dwelling adults [6, 8] emphasizing the older and frailer population in our study.

At 6 months post-fracture, the continence status had deteriorated in every fourth patient. According to a previous study, hospital-acquired UI affects up to 21% of women suffering a hip fracture [30]. Advancing age, severe functional disability, and cognitive impairment have been shown to be major risk factors for developing UI and FI during hospitalization [31].

One of the main findings of our study is the association between incontinence and functional disability and reduced mobility. In the multivariable analyses, patients with difficulties in IADL in the outpatient CGA were almost twice more likely to report UI and six times more likely to report DI while patients with difficulties in basic ADL and mobility according to the EMS scores were twice as likely to report UI and five times more likely to report DI, respectively. Furthermore, the patients with non-independent mobility at baseline had a twofold risk for DI at follow up. Similar associations have been described in prior studies for UI and for DI in a long-term care setting [9, 10, 32].

As observed in our earlier study, nutritional status deteriorated in a significant proportion of the patients during the follow up [33]. In the multivariable model, poor nutrition observed at follow up was independently associated with DI. This is not surprising as a high rate of malnutrition associated with impaired mobility and muscle strength among older hip fracture patients has been observed before, suggesting a connection between protein-energy malnutrition and sarcopenia [33]. In the present study, no significant association between decreased grip strength and UI or DI was observed. Reduced statistical power due to a large proportion of missing data in this domain may have influenced the result. Our study utilized MNA-SF only in the evaluation of nutritional status, but it has also been introduced as a screening tool for frailty with a similar cutoff value [34]. Hence, the high prevalence of poor nutrition probably also reflects a high rate of frailty in our study population.

Advancing age and cognitive disorders are well known risk factors for both UI and DI [6, 10, 32, 35]. We observed an association with age over 90 and UI, as well as diagnosis of cognitive disorder and UI. However, similar associations with DI were not observed. The limited sample of individuals with DI might partly explain the lack of association with age and cognitive disorder in these patients.

In accordance with previous observations [9, 10], patients living in a LTCF were over 2.5 times more likely to report DI at follow up as compared to those who lived at home. Pre-facture incontinence was a strong risk factor for both UI and DI at follow up in our study. DI is frequently known to precede UI in isolation [9, 10].

Depressive mood was equally common in patients with UI and DI, with one in five being affected in both groups. Depressed patients were twice as likely to report UI at follow up. In the age-adjusted univariate analysis there was also an association with DI, but it ceased to be significant in the final model. Both UI [36] and DI [6, 8] have been shown to be associated with depression in older women. Depression is a risk factor for hip fractures [37], and patients recovering from a hip fracture are at high risk of developing depression [38]. Moreover, prevalence of depression has been found to be higher in incontinent fracture patients compared to the continent patients with a fracture [29]. Screening and management of depression is an important part of CGA in older women suffering a hip fracture, since its management can positively affect the rehabilitation process, and possibly the prognosis of concurrent incontinence; thus, improving overall quality of life.

Constipation was very common in our study cohort, affecting nearly half of those with UI and 40% of those with DI. Constipation was associated with a 1.5-fold risk of UI at follow up. Constipation is a common health condition among older people with a reported prevalence of up to 50% in community-dwelling and up to 70% in populations living in LTCFs. Underlying causes are often multifactorial and related to reduced mobility, decreased liquid intake and nutrition, as well as comorbidities and polypharmacy [39]. Constipation is a known risk factor for both UI and FI [9, 39, 40]. This highlights the importance of early mobilization and sufficient nutrition and hydration of the hip fracture patients. If constipation nonetheless develops, it should be managed effectively.

The relationship with UI and falls is multidimensional, and both are indicators of frailty [11–14]. Women experience falls more often than men [41]. The risk of falls has been observed to correlate with the severity of UI [17], and DI has been associated with falls as well [16]. In our study, there was a connection with new falls after fracture in age-adjusted analysis for both UI and DI, but the association ceased to be significant in the multivariable-adjusted analyses, although the result was not far from being statistically significant for DI. The association of DI and falls warrants further studies with larger study populations.

Late removal of UC was independently associated with DI at follow up in the multivariable model and the association was nearly statistically significant for UI. The use of UCs in hospitalized older patients is widespread and often inappropriate. The use of UC increases the risk of iatrogenic urinary tract infection, which can lead to more serious complications, including bacteraemia, delirium, and death [28, 42–44]. Older women and surgical patients are at a highest risk of inappropriate UC use and thus, for adverse outcomes [42]. Prolonged use of UC or diapers during hospitalization have been shown to predict incident UI [43], and worse recovery after a hip fracture [45]. Conversely, prompt removal of UC has been shown to decrease the risk of mortality [46] and protect from decline in mobility and the need for more supported living arrangements [47] after a hip fracture.

International guidelines recommend avoiding routine UC use with surgical patients and removing the UC within 24 h postoperatively to minimize adverse effects [44]. Notably, the frailest patients with the highest risk of adverse outcomes had the highest prevalence of prolonged UC use in our study. Prompt removal of a UC postoperatively is vital in preventing complications and promoting recovery among frail hip fracture patients.

Overall, all the associations mentioned above denote underlying frailty and suggest that individuals with DI were frailer than those with UI. The connection between frailty and UI is well known [15, 48]. Associations between UI and functional disability, impaired mobility, malnutrition and cognitive impairments have also been previously demonstrated in a study of older fragility fracture patients [29]. However, there is a paucity of literature regarding DI and frailty, especially among community-dwelling adults. Our study confirms the strong association of DI with frailty in older women with hip fracture.

Importantly, frailty is believed to be at least a partially reversible condition [48]. Since hip fracture patients are generally very frail and at risk of developing incontinence, and on the other hand patients with incontinence are more prone to falls [49], a multidisciplinary treatment approach with the aim of improving patient’s mobility and nutrition might improve overall prognosis of these patients.

To our knowledge, this is the first real-world study to investigate the prevalence and associated factors of both UI and DI in a cohort of older women with hip fracture. Furthermore, our study was large and prospective in design. The outpatient CGA included well-known and standardized instruments. In addition, cognitive disorder or living in a LTCF were not among the exclusion criteria, which is an important factor in the generalizability of our findings in this patient group.

There are several limitations which should be considered when interpreting our results. Firstly, the incontinence symptoms were assessed using simple questions rather than more detailed questionnaires, or other diagnostic tools. Secondly, most of the domains of the CGA were examined only in the follow up. Thirdly, the exact duration of UC use was not known. Finally, the follow-up data was only available for 54% of the original study population. The patients who had died before follow up or could not attend the outpatient clinic were most likely the frailest and in the poorest health, which introduces the possibility of a selection bias where the prevalence of UI and especially DI is underestimated.

### Conclusions and implications

We demonstrated a high prevalence of UI and DI in older women with hip fracture in this large real-world prospective study. Both UI and DI were associated with multiple functional impairments and modifiable risk factors such as constipation, depressive mood, poor nutrition, and prolonged UC use, all of which should be targeted in orthogeriatric management and secondary falls prevention. Increased clinical awareness of incontinence and its consequences and complications in older frail hip fracture patients is paramount.

## Supplementary Information

Below is the link to the electronic supplementary material.

Supplementary file1 (DOCX 34 KB)
