# Supplementary material for: Factors associated with urinary and double incontinence in a geriatric post-hip fracture assessment in older women
Source: Aging Clin Exp Res. 2022 Jan 4;34(6):1407–18. doi: 10.1007/s40520-021-02046-z (PMC9151507; doi:10.1007/s40520-021-02046-z)
Supplement: Supplementary file 1 — Supplementary file1 (DOCX 34 KB) [file 40520_2021_2046_MOESM1_ESM.docx]

Appendix 1.

Table A1. Age-adjusted associations of baseline indicators of urinary and double incontinence after six-months follow-up among hip fracture patients (N=910) were analyzed using multinomial logistic regression. Reference group for both was no incontinence (n=343).

|  | **Urinary incontinent (n=469)** | | | | | **Double incontinent (n=98)** | | | | |
| --- | --- | --- | --- | --- | --- | --- | --- | --- | --- | --- |
|  | **Age-adjusted** | | | | | **Age-adjusted** | | | | |
|  | **n** | **(%)** | **OR** | **(95% CI)** | **p** | **n** | **(%)** | **OR** | **(95% CI)** | **p** |
| **Age** |  |  |  |  |  |  |  |  |  |  |
| 65-79 | 113 | (24) | 1.00 |  |  | 27 | (28) | 1.00 |  |  |
| 80-89 | 274 | (58) | **2.93** | **(1.82-4.71)** | **<0.001** | 53 | (54) | **2.69** | **(1.32-5.45)** | **0.006** |
| ≥ 90 | 82 | (17) | **1.82** | **(1.33-2.49)** | **<0.001** | 18 | (18) | 1.48 | (0.88-2.47) | 0.139 |
| **Fracture type** |  |  |  |  |  |  |  |  |  |  |
| Intracapsular | 276 | (59) | 1.00 |  |  | 58 | (59) | 1.00 |  |  |
| Extracapsular | 193 | (41) | 1.32 | (0.98-1.77) | 0.070 | 39 | (40) | 1.28 | (0.80-2.05) | 0.303 |
| **ASA** |  |  |  |  |  |  |  |  |  |  |
| 1-2 | 71 | (15) | 1.00 |  |  | 8 | (8) | 1.00 |  |  |
| 3-5 | 390 | (83) | 1.30 | (0.89-1.88) | 0.171 | 88 | (90) | **2.74** | **(1.26-5.98)** | **0.011** |
| **Number of regularly taken medications** |  |  |  |  |  |  |  |  |  |  |
| < 4 | 80 | (17) | 1.00 |  |  | 7 | (7) | 1.00 |  |  |
| 4-10 | 301 | (64) | 1.41 | (0.99-2.02) | 0.060 | 72 | (74) | **3.88** | **(1.71-8.80)** | **0.001** |
| > 10 | 88 | (19) | **1.80** | **(1.12-2.88)** | **0.015** | 19 | (19) | **4.47** | **(1.75-11.4)** | **0.002** |
| **Diagnosis of cognitive disorder** |  |  |  |  |  |  |  |  |  |  |
| No | 320 | (68) | 1.00 |  |  | 55 | (56) | 1.00 |  |  |
| Yes | 148 | (32) | **3.68** | **(2.46-5.49)** | **<0.001** | 43 | (44) | **6.45** | **(3.78-11.0)** | **<0.001** |
| **Mobility** |  |  |  |  |  |  |  |  |  |  |
| Independent | 267 | (57) | 1.00 |  |  | 30 | (31) | 1.00 |  |  |
| Non-independent | 201 | (43) | **2.50** | **(1.80-3.48)** | **<0.001** | 68 | (69) | **8.43** | **(5.01-14.2)** | **<0.001** |
| **Living arrangements** |  |  |  |  |  |  |  |  |  |  |
| Home | 353 | (75) | 1.00 |  |  | 45 | (46) | 1.00 |  |  |
| Institution | 112 | (24) | **2.58** | **(1.70-3.92)** | **<0.001** | 53 | (54) | **10.20** | **(5.93-17.5)** | **<0.001** |
| **MNA-SF before hip fracture** |  |  |  |  |  |  |  |  |  |  |
| Normal (12-14) | 220 | (47) | 1.00 |  |  | 29 | (30) | 1.00 |  |  |
| Poor nutrition (< 12) | 180 | (38) | **1.77** | **(1.26-2.48)** | **0.001** | 45 | (46) | **3.43** | **(2.00-5.90)** | **<0.001** |
| Not known | 69 | (15) | **0.65** | **(0.45-0.95)** | **0.024** | 24 | (25) | 1.72 | (0.94-3.14) | 0.076 |
| **Removal of urine catheter** |  |  |  |  |  |  |  |  |  |  |
| During hospital stay | 263 | (56) | 1.00 |  |  | 39 | (40) | 1.00 |  |  |
| Later | 200 | (43) | **1.46** | **(1.08-1.96)** | **0.013** | 59 | (60) | **3.02** | **(1.89-4.82)** | **<0.001** |
| **Continence before fracture** |  |  |  |  |  |  |  |  |  |  |
| Continent | 149 | (32) | 1.00 |  |  | 14 | (14) | 1.00 |  |  |
| Urinary incontinent | 282 | (60) | **6.81** | **(4.89-9.48)** | **<0.001** | 53 | (54) | **13.84** | **(7.25-26.4)** | **<0.001** |
| Double incontinent | 38 | (8) | **16.40** | **(5.73-47.0)** | **<0.001** | 31 | (32) | **143.90** | **(44.5-464)** | **<0.001** |

Results are shown by odds ratios (OR) with 95% Confidence intervals (CI). Statistically significant (p < 0.05) ORs are in **bold**. ASA, American Society of Anesthesiologists -risk score; MNA-SF, Mini Nutritional Assessment Short Form.

Appendix 2.

Table A2. Age-adjusted associations of the outpatient domains with urinary or double incontinence after 6 months follow-up among hip fracture patients (N=910) were analyzed using multinomial logistic regression. Reference group was no incontinence (n=343).

|  | **Urinary incontinent (n=469)** | | | | | **Double incontinent (n=98)** | | | | |
| --- | --- | --- | --- | --- | --- | --- | --- | --- | --- | --- |
|  | **Age-adjusted** | | | | | **Age-adjusted** | | | | |
|  | **n** | **(%)** | **OR** | **(95% CI)** | **p** | **n** | **(%)** | **OR** | **(95% CI)** | **p** |
| **Age** |  |  |  |  |  |  |  |  |  |  |
| 65-79 | 113 | (24) | 1.00 |  |  | 27 | (28) | 1.00 |  |  |
| 80-89 | 274 | (58) | **2.93** | **(1.82-4.71)** | **<0.001** | 53 | (54) | **2.69** | **(1.32-5.45)** | **0.006** |
| ≥ 90 | 82 | (17) | **1.82** | **(1.33-2.49)** | **<0.001** | 18 | (18) | 1.48 | (0.88-2.47) | 0.139 |
| **MMSE** |  |  |  |  |  |  |  |  |  |  |
| Normal (24-30) | 144 | (31) | 1.00 |  |  | 15 | (15) | 1.00 |  |  |
| Abnormal (< 24) | 308 | (66) | **1.70** | **(1.25-2.23)** | **0.001** | 71 | (72) | **4.10** | **(2.22-7.56)** | **< 0.001** |
| **IADL** |  |  |  |  |  |  |  |  |  |  |
| No difficulties (8) | 54 | (12) | 1.00 |  |  | 2 | (2) | 1.00 |  |  |
| Difficulties (0-7) | 407 | (87) | **2.56** | **(1.73 -3.77)** | **< 0.001** | 96 | (98) | **18.63** | **(4.44-78.2)** | **< 0.001** |
| **GDS-15** |  |  |  |  |  |  |  |  |  |  |
| Normal (0-6) | 345 | (74) | 1.00 |  |  | 57 | (58) | 1.00 |  |  |
| Depressed (> 6) | 98 | (21) | **2.30** | **(1.51-3.51)** | **< 0.001** | 19 | (19) | **2.73** | **(1.45-5.12)** | **0.002** |
| **EMS** |  |  |  |  |  |  |  |  |  |  |
| Normal (14-20) | 284 | (61) | 1.00 |  |  | 27 | (28) | 1.00 |  |  |
| Abnormal (< 14) | 159 | (34) | **2.93** | **(2.03-4.22)** | **< 0.001** | 55 | (56) | **11.39** | **(6.51-19.9)** | **< 0.001** |
| **TUG** |  |  |  |  |  |  |  |  |  |  |
| Normal (1-2) | 119 | (25) | 1.00 |  |  | 13 | (13) | 1.00 |  |  |
| Abnormal (3-5) | 270 | (58) | **1.77** | **(1.28-2.43)** | **< 0.001** | 40 | (8) | **2.54** | **(1.29-4.98)** | **0.007** |
| Not known | 80 | (17) | **3.11** | **(1.89-5.13)** | **< 0.001** | 45 | (46) | **17.0** | **(8.08-36.0)** | **< 0.001** |
| **Grip strength, stronger hand** |  |  |  |  |  |  |  |  |  |  |
| Normal (≥ 16 kg) | 90 | (19) | 1.00 |  |  | 10 | (10) | 1.00 |  |  |
| Abnormal (< 16 kg) | 258 | (55) | 1.24 | (0.85-1.82) | 0.266 | 46 | (47) | 1.98 | (1.00-3.93) | 0.051 |
| Not known | 121 | (26) | 0.81 | (0.56-1.17) | 0.258 | 42 | (43) | 2.35 | (1.26-4.40) | 0.008 |
| **MNA-SF** |  |  |  |  |  |  |  |  |  |  |
| Normal (12-14) | 180 | (38) | 1.00 |  |  | 12 | (12) | 1.00 |  |  |
| Poor nutrition (< 12) | 285 | (61) | **1.62** | **(1.21-2.17)** | **0.001** | 81 | (83) | **7.21** | **(3.78-13.8)** | **<0.001** |
| **Constipation** |  |  |  |  |  |  |  |  |  |  |
| No | 143 | (31) | 1.00 |  |  | 27 | (28) | 1.00 |  |  |
| Yes | 220 | (47) | **2.02** | **(1.44-2.85)** | **< 0.001** | 40 | (41) | **1.96** | **(1.12-3.44)** | **0.019** |
| Not known | 106 | (23) | 0.90 | (0.63-1.29) | 0.567 | 31 | (32) | 1.41 | (0.79-2.51) | 0.241 |
| **New falls after fracture** |  |  |  |  |  |  |  |  |  |  |
| No | 327 | (70) | 1.00 |  |  | 67 | (68) | 1.00 |  |  |
| Yes | 142 | (30) | **1.55** | **(1.12-2.17)** | **0.009** | 31 | (32) | **1.69** | **(1.02-2.80)** | **0.041** |

Results are shown by odds ratios (OR) with 95% Confidence intervals (CI). Statistically significant (p < 0.05) ORs are in **bold**. MMSE, Mini Mental State Examination; IADL, Instrumental Activities of Daily Living; GDS-15, Geriatric depression scale; EMS, Elderly Mobility Scale; TUG, Timed Up and Go -test; MNA-SF, Mini Nutritional Assessment Short Form.
